# Comparative Efficacy and Safety of Thalidomide and Sulfasalazine in Moderate to Severe Ankylosing Spondylitis: A Real-World Study From Bangladesh

**DOI:** 10.7759/cureus.86211

**Published:** 2025-06-17

**Authors:** A F M Mahbubul Alam, Mohammad Abul Kalam Azad, M Masudul Hassan, Sajeda Islam, Md Nazrul Islam, Minhaj Rahim Choudhury, Syed Atiqul Haq

**Affiliations:** 1 Rheumatology, Mymensingh Medical College, Mymensingh, BGD; 2 Rheumatology, Bangladesh Medical University, Dhaka, BGD; 3 Physical Medicine and Rehabilitation, Mugda Medical College, Dhaka, BGD; 4 Rheumatology, Bangabandhu Sheikh Mujib Medical University, Dhaka, BGD; 5 Rheumatology, Green Life Center for Rheumatic Care and Research, Dhaka, BGD

**Keywords:** ankylosing spondylitis, back pain, spondyloarthritis, sulfasalazine, thalidomide

## Abstract

Background: The present study aimed to determine the efficacy and safety of thalidomide compared to sulfasalazine (SSZ) in moderate to severe cases of ankylosing spondylitis (AS) in Bangladesh.

Methods: This experimental study was conducted from January 2017 to December 2018 in the Department of Rheumatology, Bangabandhu Sheikh Mujib Medical University (BSMMU), Dhaka, Bangladesh. The experimental study included 93 adult patients with moderate to severe AS (47 received thalidomide, and 46 received SSZ). Change in disease status was considered an efficacy endpoint, while the prevalence of adverse events was considered a safety endpoint.

Results: Thalidomide showed better efficacy compared to SSZ in terms of disease activity index (Bath Ankylosing Spondylitis Disease Activity Index) (2.84 vs. 4.49, p < 0.001). Secondary endpoints such as functional index (Bath Ankylosing Spondylitis Functional Index) (2.32 vs. 3.34, p = 0.021), patients’ global assessment (2.92 vs. 4.11, p < 0.001), physician global assessment (2.67 vs. 3.60, p < 0.001), spinal pain score (2.52 vs. 3.31, p = 0.025), swollen joint count (0.05 vs. 0.40, p < 0.001), morning stiffness (2.08 vs. 3.27, p < 0.001), erythrocyte sedimentation rate (28.17 mm vs. 34.25 mm, p = 0.023), and C-reactive protein (9.57 mg/l vs. 21.34 mg/l, p = 0.027) were also better in the thalidomide group. Moreover, a significantly higher number of patients achieved 40% improvement in the thalidomide group compared to the SSZ group (90% vs. 10%, p < 0.001). Pedal edema was the most common adverse event (16, 46%) in the thalidomide group, followed by somnolence (13, 37%), drowsiness (12, 34%), dry mouth (11, 31%), and skin rash (11, 31%). On the other hand, anorexia (17, 48%), headache (15, 43%), and gastric irritation (10, 28%) were common adverse events in the SSZ group.

Conclusion: Despite a few mild side effects, thalidomide is effective and safe in comparison with SSZ for treating patients with moderate to severe AS for short-term assessment.

## Introduction

Ankylosing spondylitis (AS) is a chronic inflammatory condition most commonly affecting the sacroiliac joint. In its extreme form, AS can lead to spinal bone protrusion, paraspinal soft tissue damage, and bony fusion of vertebral joints [1]. Characteristic clinical manifestations of AS include spinal stiffness resulting in back pain and limited spinal mobility [2]. The peripheral joint involvement of AS may also occur, usually monoarticular or oligoarticular, and affects mainly the lower limbs [3]. The prevalence of this disease ranges from 0.9% to 1.4% of the adult population, with a significant predominance in young men, as about 80% of patients with AS develop their first symptoms at an age younger than 30 years [4].

Treatment options for AS are limited. It is reported that nonsteroidal anti-inflammatory drugs (NSAIDs), disease-modifying antirheumatic drugs (DMARDs), and biologics, particularly tumor necrosis factor-alpha (TNF-α) inhibitors, are commonly used for managing AS globally [5]. However, it is reported by Escalas et al. in 2010 [6] that the effectiveness of standard doses of NSAIDs is limited to patients with mild disease without spinal fusion or joint destruction, leaving the pain in patients with severe AS often refractory to NSAIDs. Similarly, the use of DMARDs, especially sulfasalazine (SSZ) [7], is not fully supported by robust evidence regarding its effect on reducing pain, disease activity, radiographic progression, or improving physical function and spinal mobility in treating AS. These limitations underscore the need for more effective and cost-efficient alternatives. TNF-α is a pro-inflammatory cytokine that is involved in the pathogenesis of AS. It is also reported by Braun and Sieper [2] that the abundance of tumor necrosis factor (TNF) messenger RNA has been detected in these patients' synovial biopsies from sacroiliac joints. Hence, TNF-α blocking agents like infliximab, etanercept, and adalimumab have emerged as an effective therapy for almost all features of AS [8].

However, most of these drugs are not cost-effective for patients from low- and middle-income countries like Bangladesh. In this context, Paumgartten [9] reported that thalidomide, a potential anti-TNF agent, can be considered a cost-effective alternative for treating AS. A meta-analysis by Yao et al. [10] has shown that the disease condition of AS in patients treated with thalidomide significantly improved compared with conventional therapy.

Wang et al. (2022) [11] reported that the time of morning stiffness, disease activity, C-reactive protein (CRP) level, and other related symptoms and indices of AS were also significantly optimized in patients treated with thalidomide, as also reported by Raychaudhuri and Deodhar [12]. This underscores the potential of thalidomide as a cost-effective and optimistic treatment for AS. Thalidomide would be a good option for treating moderate and severe cases of AS in a low-resource country like Bangladesh, considering its cost-effectiveness and easy availability.

This study aimed to compare the short-term efficacy and safety of thalidomide versus sulfasalazine in adult patients with moderate to severe AS in a real-world clinical setting in Bangladesh.

## Materials and methods

Study design and setting

This open-label randomized trial was conducted from January 2017 to December 2018 in the Department of Rheumatology of Bangabandhu Sheikh Mujib Medical University (BSMMU), Dhaka, Bangladesh. Ethical clearance was obtained from the Institutional Review Board (IRB) of BSMMU (Approval number: BSMMU/2013/1638), and the study was also registered in ClinicalTrials.gov (NCT06985134).

Participants

The present study included patients aged >18 years with inflammatory low back pain fulfilling the Modified New York criteria (2014) [12] for moderate to severe AS. The criteria include radiographic sacroiliitis (at least grade II bilaterally or grade III unilaterally) along with clinical signs, such as inflammatory back pain for at least three months, limitation of lumbar spine in sagittal and frontal planes, or chest expansion decreased relative to normal values for age and sex. Exclusion criteria were non-inflammatory back pain or back pain due to inflammatory causes other than AS, failure to confirm a washout period of four weeks, those who were on DMARDs, allergy to thalidomide, sulfasalazine, and NSAIDs, known to have kidney diseases or cardiac disease, active peptic ulcer disease, and pregnancy.

The sample size for the present study was calculated using the OpenEpi software according to the Kelsey method [13], considering a 95% significance level, 80% power, and a favorable clinical outcome in the thalidomide group of 80% and 53% in the SSZ group [14]. These estimations provided that 48 patients in each group would be sufficient for the present study. A total of 93 patients, 47 patients in the thalidomide group and 46 patients in the SSZ group, were included in the study at baseline. Among them, 70 patients (35 in each group) completed the follow-up in the sixth month.

All the enrolled patients were randomly allocated into two groups following even and odd enrollment serial numbers. Even-number cases were defined as the intervention group (thalidomide) and odd-number cases as the control (sulfasalazine). Neither the patients nor the physicians were blinded in the present study. The manufacturer of sulfasalazine (salazine) is Opsonin Pharma Ltd. (Dhaka, Bangladesh), and the manufacturer of thalidomide (thalimide) is Beacon Pharmaceuticals (Dhaka, Bangladesh).

Intervention and follow-ups

The thalidomide group of patients with AS received cap thalidomide 100 mg/day orally 12 hourly for the first 15 days and then 200 mg/day at bedtime, one hour after a meal with plenty of water. An NSAID in full doses, along with therapeutic exercise, was advocated in each patient.

On the other hand, the SSZ group of patients with AS received sulfasalazine tablets at a dose of 500 mg/day orally after a meal for the first seven days, then 500 mg twice daily for the next seven days. It was advocated that each patient reach the recommended dose of 2 grams daily. An NSAID in full dose, along with therapeutic exercise, was also advocated.

**Figure 1:**
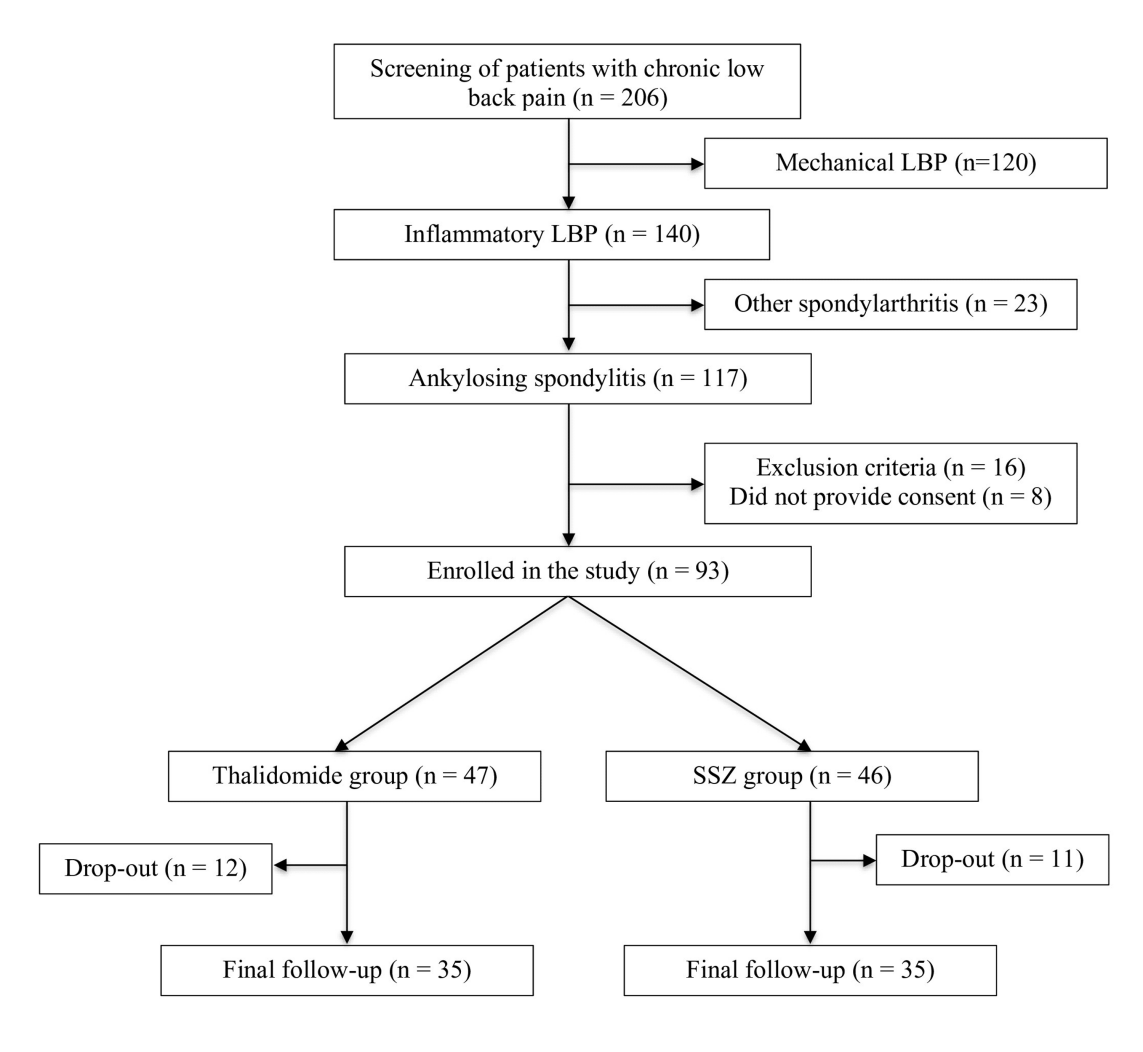
Patient enrolment and follow-up flowchart. LBP: low back pain; SSZ: sulfasalazine.

The patients were evaluated for clinical and laboratory parameters related to AS at baseline during enrollment. Subsequent follow-ups were done at the end of the first, second, fourth, and sixth months (Figure 1).

Endpoints

The study's primary efficacy endpoint was the change in the Bath Ankylosing Spondylitis Disease Activity Index (BASDAI) score at the end of the sixth month from baseline.

Secondary efficacy endpoints included the change in the score of Bath Ankylosing Spondylitis Functional Index (BASFI), Bath Ankylosing Spondylitis Metrology Index (BASMI), patients’ global assessment, physician global assessment, spinal pain score, swollen joint count, morning stiffness, and Maastricht Ankylosing Spondylitis Enthesitis Score (MASES). Other secondary endpoints were the proportion of patients achieving the Assessment of Spondyloarthritis International Society (ASAS)-20 and ASAS-40 improvement scores of AS.

The ASAS-20 [15] improvement criteria are defined as improvement of ≥20% and > one unit in at least three domains on a scale of 10 and no worsening of ≥20% and > one unit in the remaining domain on a scale of 10. The domains are patients’ global assessment, spinal pain, function (BASFI), and inflammation.

ASAS-40 improvement criteria are defined as improvement of ≥40% and > two units in at least three domains on a scale of 10 and no worsening in the remaining domain [15]. It is also reported by the Ankylosing Spondylitis Disease Activity Score (ASDAS) [15].

Data collection procedure

After screening the patients for inclusion and exclusion criteria, informed written consent for participation in the study was obtained from those who met the inclusion criteria. After that, the enrolled patients were interviewed and assessed clinically for baseline data collection. Relevant laboratory investigations and a record review of the patients were also carried out. A semi-structured case record form was used for data collection. The detailed medical history of the patients was obtained, and initial symptoms were noted chronologically from the onset. Disease activity index, which included BASDAI, BASFI, BASMI, swollen joint, MASES, and laboratory investigations (ESR, CRP), monitoring parameters for drug toxicity (hemoglobin %, alanine aminotransferase, serum creatinine) were recorded at baseline and each subsequent follow-up visit.

Operational definition

Moderate to severe AS was defined as the BASDAI score >40 (range: 0 to 100) and physician visual analog scale >40 (range: 0 to 100), as well as clinical evidence of symptomatic inflammatory low back pain and painful swollen peripheral joints [16].

The BASDAI is a 10-point linear scale, with 1 being no problem and 10 being the worst problem. It consists of six questions of five significant symptoms of AS: fatigue, spinal pain, joint pain/swelling, areas of localized tenderness (also called enthesitis or inflammation of tendons or ligaments), morning stiffness duration, and morning stiffness severity. The index results in a final 0-10 BASDAI score. The higher the score, the more severe the patient’s disease activities [17].

The BASFI is a 10-item index of daily functional activities completed on a numerical rating scale from 0 to 10. The higher the BASFI score, the more severe the functional limitation due to AS [17].

The BASMI reflects axial mobility through five clinical measurements: chest expansion, modified Schober test, tragus to wall, cervical rotation, lateral spinal flexion, and inter-malleolar distance. The higher the BASMI score, the more severe the patient’s limitation of movement due to their AS [17].

The MASES is used to assess enthesitis. This scale records tenderness on examination as either present or absent (0) after a firm palpation at a pressure of approximately 4 kg/cm2 with the pulp of the thumb at 13 sites: the bilateral first and seventh costochondral joints, the anterior and posterior superior iliac spines, the iliac crests, the fifth lumbar spinous process, and the proximal insertion of the Achilles tendon. Overall score ranges 0 to 13 [1].

Statistical analysis

After collecting information, all data were checked, verified for consistency, and entered using Microsoft Excel (Microsoft Corporation, Redmond, WA). Statistical analysis was carried out using STATA version 17.0 (StataCorp LLC, College Station, TX). Descriptive statistics like percentage, mean, and standard deviation of different variables were used. A t-test measured the comparison of quantitative variables among the groups and between groups. Categorical comparison was performed using the chi-squared test. A p-value was regarded as statistically significant if it was <0.05.

## Results

Baseline characteristics

A total of 93 patients with moderate to severe AS were included in the present study (47 in the thalidomide group and 46 in the SSZ group). The average age of the patients in the thalidomide group was 31 (SD: 10.3) years, and in the SSZ group, it was 28.6 (SD: 8.0) years. Most patients in both groups were male (97.8% in the thalidomide group and 82.6% in the SSZ group). Detailed sociodemographic characteristics of the patients are described in Table 1.

**Table 1:** Demographic characteristics of enrolled patients of both study groups (n = 93). SSZ: sulfasalazine; MTX: methotrexate; NSAIDs: nonsteroidal anti-inflammatory drugs; SD: standard deviation; CRP: C-reactive protein; ESR: erythrocyte sedimentation rate; BDT: Bangladeshi Taka; BASDAI: Bath Ankylosing Spondylitis Disease Activity Index; BASFI: Bath Ankylosing Spondylitis Functional Index; BASMI: Bath Ankylosing Spondylitis Metrology Index; MASES: Maastricht Ankylosing Spondylitis Enthesitis Score. * Test = unpaired student t test; ** test = x2 test; *** x2 value. Values in parentheses were expressed as a percentage.

Characteristics	Thalidomide (n = 47)	SSZ (n = 46)	T-value	p-value*
*Age (years), mean (±SD)	31.00 (±10.32)	28.60 (±8.03)	911.56	0.214
Sex	---	-----	---	---
Male	46 (97.87)	38 (82.61)	6.20***	0.0128
Female	1 (2.13)	8 (17.39)		
Educational status**	-----	-------	-----	----
Illiterate to primary	16 (34.0)	20 (43.4)	4.99***	0.0225
Secondary/higher secondary	18 (38.2)	19 (41.3)		
Graduate	9 (19.1)	7 (15.2)		
Post-graduate	4 (8.5)	0 (0.00)		
Occupation**	------	------	---	-----
Service holder	13 (27.6)	14 (30.4)	3.31***	0.0688
Business	5 (10.6)	2 (4.3)		
Student	11 (23.4)	9 (19.5)		
Housewife	1 (2.12)	4 (8.6)		
Others	17 (14.9)	17 (10.9)		
Marital status**	------	-----	----	---
Married	25 (53.1)	22 (47.8)	0.2677***	0.6049
Unmarried	22 (46.8)	24 (52.1)		
Monthly income**	------	----	-----	----
Low (BDT 12500 to 20000)	16 (34.04)	15 (32.61)	0.2864***	00.5925
Middle (BDT 20000 to 40000)	19 (40.43)	17 (36.96)		
High (>BDT 40000)	12 (25.53)	14 (30.43)		
Disease duration (months), mean (±SD)	59.71 (±39.88)	69.51 (±47.23)	1.0815	0.26
Disease status	----	-----	-----	-----
BASDAI (0–10), mean (±SD)	6.75 (±1.31)	6.68 (±1.29)	0.22	0.784
BASFI (0–10), mean (±SD)	6.41 (±2.19)	5.59 (±2.36)	1.73	0.085
BASMI (0–10), mean (±SD)	3.12 (±2.41)	2.78 (±2.89)	0.61	0.538
Patient’s global assessment (0–10), mean (±SD)	7.82 (±1.72)	7.50 (±1.68)	.09	0.353
Physician global assessment (0–10), mean (SD)	7.91 (±1.36)	7.54 (±1.42)	0.07	0.205
Spinal pain (0–10), mean (SD)	7.12 (±2.00)	6.59 (±1.79)	1.19	0.183
Swollen joint count (0–44), mean (SD)	1.74 (±1.89)	1.97 (±1.93)	0.58	0.558
Morning stiffness (0–10), mean (SD)	6.93 (±2.03)	6.44 (±1.82)	1.22	0.224
MASES (0–13), mean (SD)	3.40 (±3.66)	2.32 (±2.59)	1.63	0.102
ESR (mm) in the first hour, mean (SD)	71.12 (±32.41)	65.65 (±28.58)	0.86	0.397
CRP (mg/l), mean (SD)	66.51 (±60.88)	54.14 ±43.97)	1.11	0.264
Drugs taken	------	-----	---	----
Indomethacin	19 (40.43)	20 (43.48)	---	0.45
Diclofenac/aceclofenac	26 (55.32)	28 (60.87)	-----	0.67
Etoricoxib	7 (14.89)	6 (13.04)	----	0.71
Naproxen	10 (21.28)	8 (17.39)	----	0.628
Other NSAIDs	6 (12.77)	5 (10.87)	-----	0.39
MTX	14 (29.79)	17 (36.96)	-----	0.102
Prednisolone	18 (38.30)	16 (34.78)	----	0.737
Duration of drug intake (months), mean (SD)	-------	------	-----	-------
NSAIDs	26.45 (±17.34)	28.67 (±19.93)	0.57	0.453
MTX	17.75 (±17.86)	15.78 (±14.88)	2.78	0.183
Prednisolone	18.12 (±20.67)	19.45 (±21.37)	0.30	0.685

At baseline, the disease status of the patients with ankylosing spondylitis, as measured by various parameters including BASDAI, BASFI, BASMI, patients’ global assessment, physician’s global assessment, spinal pain, number of swollen joints, morning stiffness, and MASES, was found to be quite similar in both the thalidomide and SSZ groups (p-value > 0.05). Both groups also exhibited similar laboratory parameters, such as ESR and CRP (Table 1).

All the patients were using at least one NSAID before enrollment. In both groups, the most frequently used NSAIDs were diclofenac, aceclofenac, indomethacin, and naproxen. Almost one-third of the patients were taking methotrexate. Corticosteroids like prednisolone were utilized in nearly 38% of the patients in the thalidomide group and 35% in the SSZ group (Table 1).

Efficacy endpoints

A total of 70 patients completed the six-month follow-up (35 in both groups). Of these, 40 had predominantly axial AS (20 in both groups), and 30 had predominantly peripheral AS (15 in both groups).

**Figure 2:**
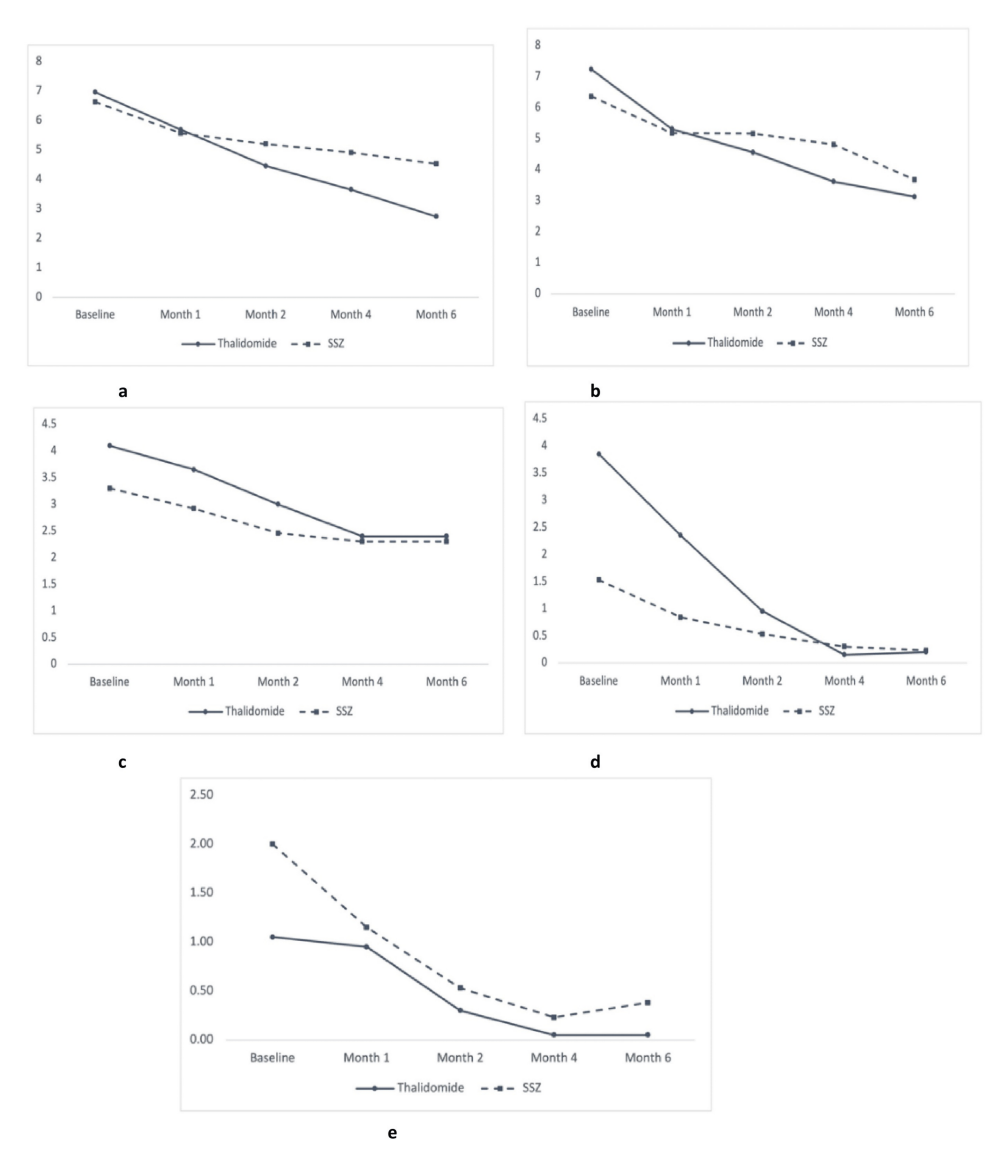
Line graphs showing evolution in clinical outcomes in both study groups in patients with ankylosing spondylitis (n = 70). (a) Bath Ankylosing Spondylitis Disease Activity Index (BASDAI). (b) Bath Ankylosing Spondylitis Functional Index (BASFI). (c) Bath Ankylosing Spondylitis Metrology Index (BASMI). (d) Maastricht Ankylosing Spondylitis Enthesitis Score (MASES). (e) Swollen joint count. The X axis shows the duration of follow-up, and the Y axis shows the disease activity status of the study subjects. SSZ: sulfasalazine.

**Figure 3:**
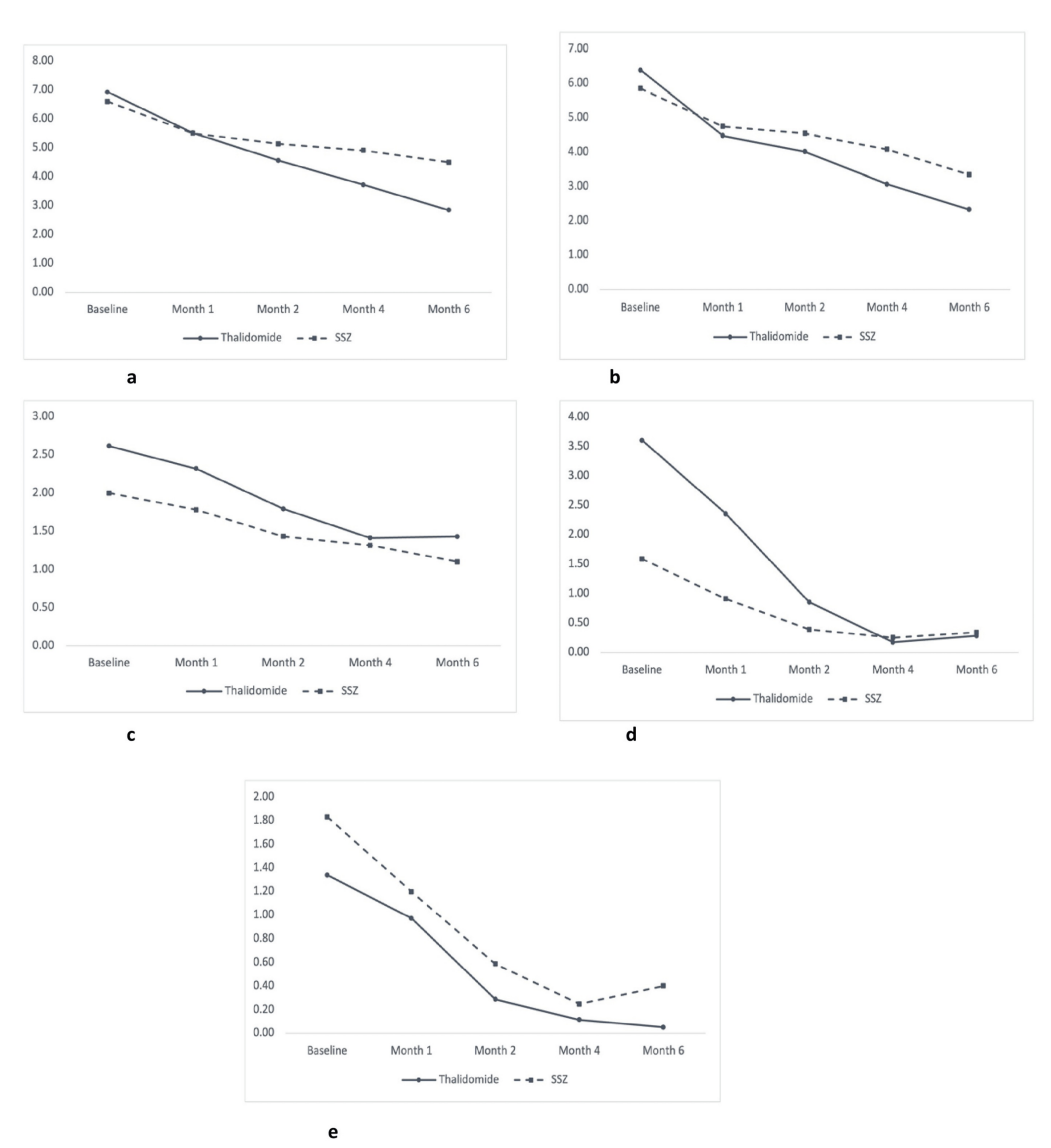
Line graphs showing evolution in clinical outcomes in both study groups in patients with predominant axial ankylosing spondylitis (n = 40) (a) Bath Ankylosing Spondylitis Disease Activity Index (BASDAI). (b) Bath Ankylosing Spondylitis Functional Index (BASFI). (c) Bath Ankylosing Spondylitis Metrology Index (BASMI). (d) Maastricht Ankylosing Spondylitis Enthesitis Score (MASES). (e) Swollen joint count. The X axis shows the duration of follow-up, and the Y axis shows the disease activity status of the study subjects. SSZ: sulfasalazine.

**Figure 4:**
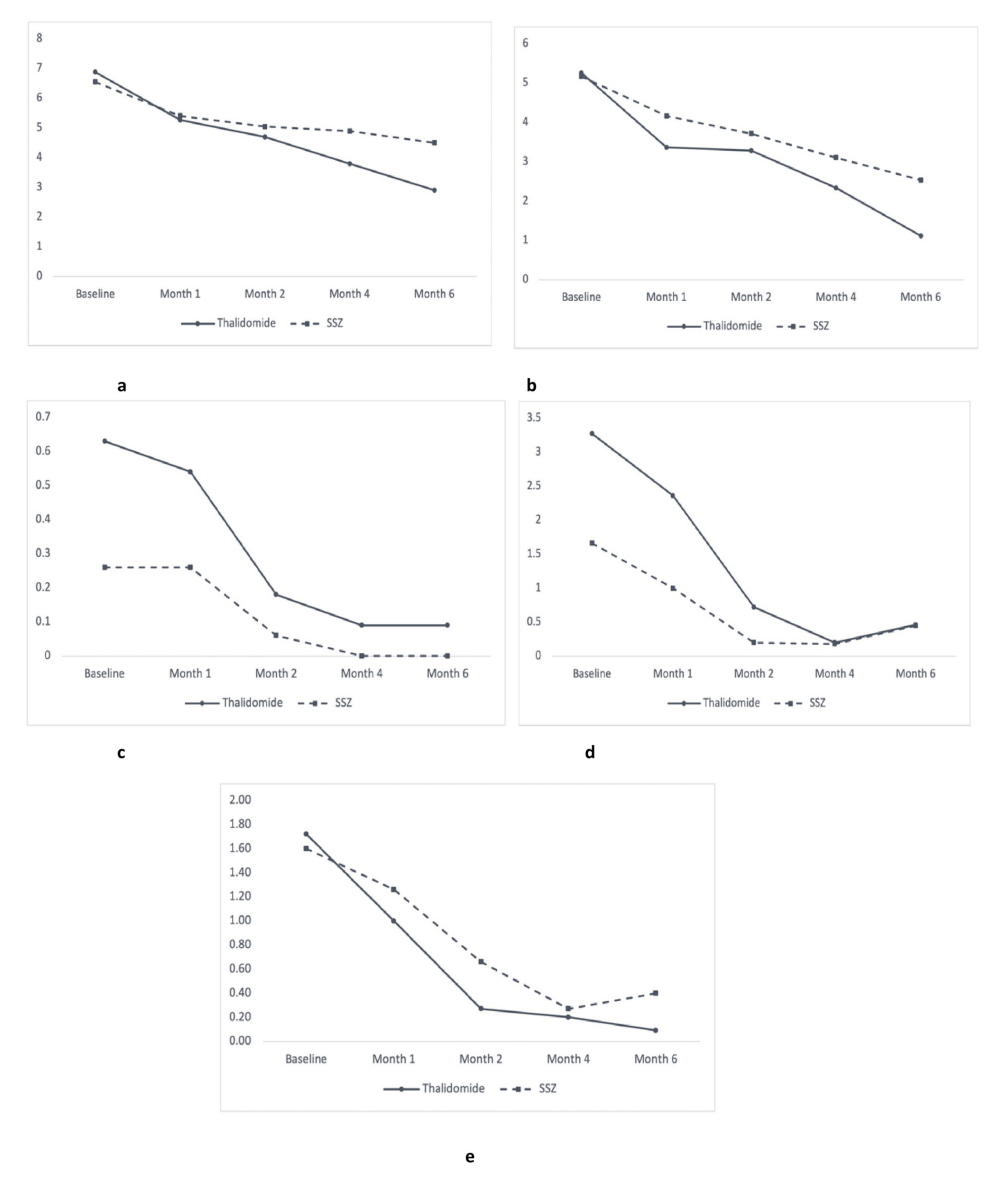
Line graphs showing evolution in clinical outcomes in both study groups in patients with predominant peripheral ankylosing spondylitis (n = 30) (a) Bath Ankylosing Spondylitis Disease Activity Index (BASDAI). (b) Bath Ankylosing Spondylitis Functional Index (BASFI). (c) Bath Ankylosing Spondylitis Metrology Index (BASMI). (d) Maastricht Ankylosing Spondylitis Enthesitis Score (MASES). (e) Swollen joint count. The X axis shows the duration of follow-up, and the Y axis shows the disease activity status of the study subjects. SSZ: sulfasalazine.

Changes in disease status of AS measured by BASDAI score, BASFI score, BASMI score, MASES score, and swollen joint count in overall patients with AS, patients with predominantly axial AS, and patients with predominantly peripheral AS were measured during different follow-ups, which are demonstrated in Figures 2-4, respectively.

At the end of six months, we observed that there was a significant difference between thalidomide and SSZ groups in terms of mean BASDAI score, which was the primary endpoint of the study (2.84 in the thalidomide group vs. 4.49 in the SSZ group, p-value <0.001). Significant improvements were also observed in other secondary endpoints in the thalidomide group compared to the SSZ group, including BASFI score (2.32 vs. 3.34, p-value 0.021), patient's global assessment score (2.92 vs. 4.11, p-value <0.001), physician global assessment score (2.67 vs. 3.60, p-value <0.001), spinal pain score (2.52 vs. 3.31, p-value 0.025), swollen joint count (0.05 vs. 0.40, p-value <0.001), and morning stiffness score (2.08 vs. 3.27, p-value <0.001). Laboratory parameters also significantly improved in patients receiving thalidomide compared to the patients receiving SSZ (ESR = 28.17 mm vs. 34.25 mm, p-value = 0.023; and CRP = 9.57 mg/l vs. 21.34 mg/l, p-value = 0.027) (Table 2).

**Table 2:** Disease status in both study groups at sixth month (n = 70). SSZ: sulfasalazine; PGA: patients' global assessment; Ph GA: physician global assessment; SD: standard deviation; BASDAI: Bath Ankylosing Spondylitis Disease Activity Index; MS: morning stiffness; BASFI: Bath Ankylosing Spondylitis Functional Index; BASMI: Bath Ankylosing Spondylitis Metrology Index; MASES: Maastricht Ankylosing Spondylitis Enthesitis Score. * Paired t-test.

Clinical variables	Thalidomide (n = 35)	SSZ (n = 35)	T score	P-value
BASDAI (0-10), mean (±SD)	2.84 (±0.99)	4.49 (±0.74)	7.82	<0.001
BASFI (0-10), mean (±SD)	2.32 (±1.08)	3.34 (±2.02)	2.63	0.021
BASMI (0-10), mean (±SD)	1.43 (±1.21)	0.10 (±0.90)	5.22	0.37
PGA (0-10), mean (±SD)	2.92 (±1.13)	2.92 (±1.13)	2.99	<0.001
Ph GA (0-10), mean (±SD)	2.67 (±0.96)	3.60 (±1.01)	3.95	<0.001
Spinal pain (0-10), mean (±SD)	2.52 (±1.42)	3.31 (±1.32)	2.41	0.025
Swollen joint count (0-44), mean (±SD)	0.05 (±0.23)	0.40 (±0.49)	14.57	<0.001
MS (0-10), mean (±SD)	2.08 (±1.39)	3.27 (±1.26)	3.75	<0.001
MASES (0-13), mean (±SD)	0.28 (±0.45)	0.34 (±0.48)	0.54	0.61

**Figure 5:**
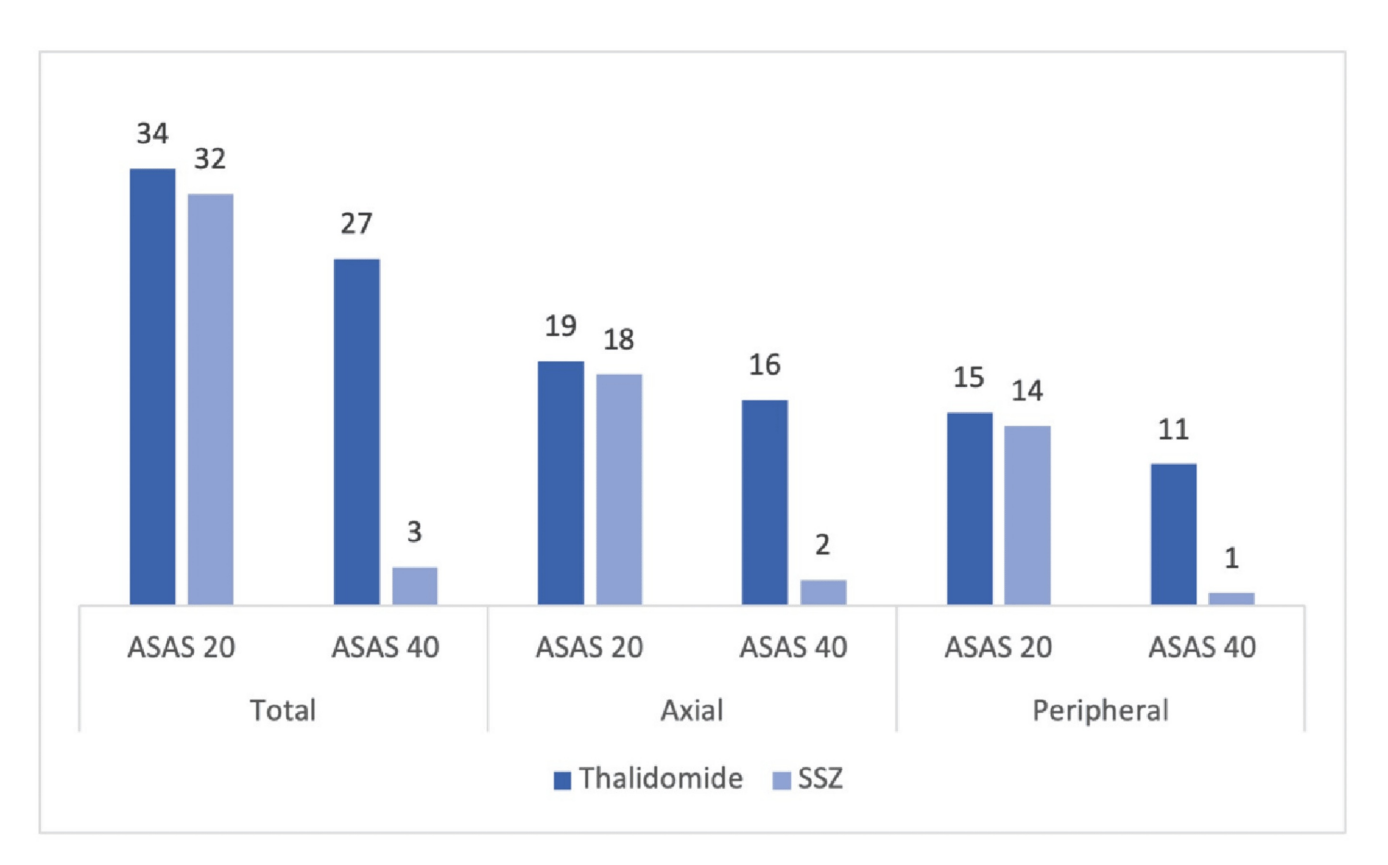
Patients with ankylosing spondylitis achieving ASAS-20 and ASAS-40 at sixth months (n = 70). Total ASAS-20 vs. ASAS-40 (p < 0.003), axial ASAS-20 vs. ASAS-40 (p = 0.007), and peripheral ASAS-20 vs. ASAS-40 (p = 0.01) was calculated by chi-square test. SSZ: sulfasalazine; ASAS: Assessment of Spondyloarthritis International Society.

Overall, a total of 34 patients with AS out of 35 in the thalidomide group and 32 patients out of 35 in the SSZ group achieved an ASAS-20 improvement score at the end of six months. However, it was not statistically significant. On the other hand, 27 patients in the thalidomide group and three in the SSZ group achieved an ASAS-40 improvement score at the end of six months, which was statistically significant (p-value <0.001). In predominant axial AS patients of the thalidomide group, ASAS-20 and ASAS-40 scores were achieved by 95% and 80% of patients, respectively. In the sulfasalazine group, ASAS-20 and ASAS-40 scores were achieved by 90% and 10% of patients, respectively. In patients with predominant peripheral AS, the response to thalidomide at 20 and 40% was completed by 100% and 73%, respectively, and in the sulfasalazine group, it was 93% and 7%, respectively (Figure 5).

Safety endpoints

Among the patients with AS in the thalidomide group, pedal edema was the most common adverse event reported in almost 46% of the patients. Other commonly reported adverse events were somnolence (37%), drowsiness (34%), dry mouth (31%), and skin rash (31%). All the adverse events were mild, and no severe adverse event was reported. On the other hand, anorexia, headache, and gastric irritation were common adverse events among patients in the SSZ group, reported in almost 48%, 43%, and 28% of the patients, respectively (Table 3).

**Table 3:** Adverse events of thalidomide and SSZ in patients with ankylosing spondylitis (n = 70). SSZ: sulfasalazine.

Adverse events	n	%
Thalidomide		
Dry mouth	11	31.43
Constipation	8	22.86
Headache	3	8.57
Dizziness	9	25.71
Somnolence	13	37.14
Drowsiness	12	34.29
Skin rash	11	31.43
Pedal edema	16	45.71
SSZ		
Anorexia	17	48.57
Headache	15	42.86
Nausea	9	25.71
Gastric irritation	10	28.57
Skin rash	1	2.86

## Discussion

Recently, Osman and Maksymowych [8] reported that anti-TNF-based treatment has demonstrated better results in patients with AS. However, the high price remained one of the significant barriers to using these agents in resource-constrained countries like Bangladesh.

In this context, thalidomide, a low-cost therapeutic agent, was effective in severe active AS cases for its anti-TNF activity [18]. Hence, the present study aimed to evaluate the comparative efficacy and safety of thalidomide compared to sulfasalazine in patients with moderate to severe AS. The results of our study demonstrated that patients receiving thalidomide for management of AS had a better improvement in disease status of AS compared to the patients receiving SSZ in terms of disease activity index (BASDAI), functional index (BASFI), patients’ global assessment, physician global assessment, spinal pain score, swollen joint count, and morning stiffness. Moreover, ESR and CRP levels, which are considered as indicators of disease activity of AS, also significantly decreased in patients receiving thalidomide compared to the patients receiving SSZ. Our study also demonstrated the better efficacy of thalidomide compared to SSZ in both axial and peripheral AS subgroups.

Several clinical trials have shown findings similar to those of our study. A one-year open-label clinical trial of thalidomide on patients with AS reported that 80% of the patients receiving thalidomide achieved >20% improvement in primary indices of disease activity, namely, BASDAI, BASFI, overall body pain, spinal pain, patients’ global assessment, and physician global assessment [18]. A recently published meta-analysis by Choudhury et al. [19], including 1471 patients with AS from China, reported that the effectiveness of thalidomide alone and combined with other drugs was significantly higher than that of the control group. Patients receiving thalidomide treatment showed a significant improvement in AS disease activity indices [11]. Though evidence is scarce regarding the efficacy of thalidomide in patients with AS from Bangladesh, a clinical study reported that this drug improved disease activity in terms of BASDAI, BASFI, and BASMI, as found in our study [19]. However, that was a single-arm study without any comparison group, which limits the robustness of the evidence.

In the present study, SSZ was used for comparison as it is commonly used to manage AS in Bangladesh. A previous study conducted on patients with AS from Bangladesh reported that all the patients received SSZ during treatment [19]. Almost 20% of the patients in our study also received SSZ before enrollment. Though most clinical studies failed to demonstrate any significant role of SSZ in treating AS, few studies reported a beneficial effect of this drug on disease activity, especially in patients with predominantly peripheral AS [20,21].

In our study, typical side effects of thalidomide were pedal edema, somnolence, drowsiness, dry mouth, skin rash, and constipation. However, the high frequency of pedal edema may also be attributed to concomitant use of NSAIDs, mainly indomethacin and etoricoxib. In a clinical study by Huang et al. (2002) [18], the significant side effects were slight drowsiness and dry mouth, which resolved after four weeks of continued therapy. Wei et al. (2003) [22] also observed dry mouth, constipation, and dizziness as common side effects in patients receiving thalidomide. Findings of this series are comparable to those of other studies. On the other hand, in patients receiving SSZ, anorexia, headache, and gastric irritation were commonly reported adverse events. Similar findings were also noted in several studies [23,24].

In our study, the dropout rate was considerably high, 12 (25.5%) patients in the thalidomide group and 11 (23.9%) patients in the SSZ group. In the thalidomide group, nine patients dropped out as they failed to purchase the drug, and in the SSZ group, one patient developed neuropathy and one developed hypersensitivity reactions. However, a high dropout rate was also reported in a previous trial of thalidomide on patients with AS. In Zhu et al.'s (2010) [25] study, 32 patients (13.8% out of 232 enrolled patients) withdrew due to adverse events.

Our study provided evidence regarding the safety and efficacy of thalidomide in patients with moderate to severe AS. However, it has several limitations. Firstly, it was an open-label study, which might increase the risk of biased reporting and interpretation of results. Moreover, the study's follow-up period was too short to demonstrate long-term robust evidence of the trial drug's efficacy and safety. Finally, the patients' high dropout rate might affect the statistical strength of the study findings.

## Conclusions

In conclusion, thalidomide showed significantly higher efficacy than SSZ in improving disease status in patients with moderate to severe AS regarding disease activity, functionality, spinal pain, swollen joint count, and morning stiffness. Moreover, thalidomide was found safe to use in patients with AS despite some mild and short-term adverse events.
